# Impact of Breast Cancer Awareness Month on Public Interest in the United States between 2012 and 2021: A Google Trends Analysis

**DOI:** 10.3390/cancers14102534

**Published:** 2022-05-21

**Authors:** Yoshito Nishimura, Jared D. Acoba

**Affiliations:** 1 Department of Medicine, John A. Burns School of Medicine, University of Hawai’i, Honolulu, HI 96813, USA; jacoba@hawaii.edu; 2 Department of General Medicine, Okayama University Graduate School of Medicine, Dentistry and Pharmaceutical Sciences, Okayama 7008558, Japan

**Keywords:** Breast Cancer Awareness Month, breast cancer, Google Trends, trend analysis

## Abstract

**Simple Summary:**

In this study, we quantified public awareness regarding breast cancer associated with Breast Cancer Awareness Month (BCAM) in October using Google Trends data and a joinpoint regression analysis. We analyzed the impact of BCAM, Lung Cancer Awareness Month (LCAM), and Prostate Cancer Awareness Month (PCAM) on public awareness of the top three most common cancers in the U.S. from 2012 to 2021 using the relative search volume of Google Trends. The results imply that BCAM has successfully improved the public awareness of breast cancer in the U.S., while LCAM and PCAM had no impact on the awareness of lung and prostate cancers. BCAM could serve as a good example for organizations working on health observances or awareness campaigns.

**Abstract:**

Breast Cancer Awareness Month (BCAM) has a long history of over 30 years, established in 1985 to occur every October, and the National Breast Cancer Foundation now leads the operation. There have been no studies to evaluate the impact of the BCAM on public awareness of breast cancer. We analyzed the impact of BCAM on public awareness of breast cancer in the U.S. from 2012 to 2021 using the relative search volume (RSV) of Google Trends as a surrogate. We also analyzed the impact of Lung Cancer Awareness Month (LCAM) and Prostate Cancer Awareness Month (PCAM) on public awareness of lung and prostate cancer, respectively, to see differences in their effectiveness among the health observances for the top three most common cancers in the U.S. We performed a joinpoint regression analysis to identify statistically significant time points of a change in trend. There were joinpoints around BCAM for “Breast cancer” every year from 2012 to 2021, with a significant increase in the weekly RSVs from 21.9% to 46.7%. Except for 2013 and 2015 for “Lung cancer”, when significant increases in the RSV at 1.8% and 1.2% per week were observed around LCAM, no joinpoints were noted around LCAM or PCAM. These results imply that BCAM has successfully improved the public awareness of breast cancer in the U.S. compared to other representative health observances, likely due to the effective involvement of non-medical industries, influencers affected by breast cancer, and an awareness symbol.

## 1. Introduction

Breast cancer is the most common type of malignancy in the United States (U.S.), with 281,550 new female cases diagnosed and 43,600 deaths in 2020 [1]. Lung cancer and prostate cancer were marked as the second and third most common cancers in the U.S. in 2020. Given the increasing disease burden of cancers, prevention and early detection of the disease are considered crucial.

Due to the increasing prevalence and mortality related to cancers, public health authorities and organizations working on cancers have taken various measures, including establishing health observances. Currently, there are a number of national health observances recognized by the U.S. Department of Health and Human Services to call on the general public to increase awareness of specific health topics [2]. Breast Cancer Awareness Month (BCAM) has a long history of over 30 years. In 1985, the American Cancer Society and the American Academy of Family Physicians, in collaboration with multiple stakeholders, established BCAM to occur every October. The National Breast Cancer Foundation now leads the BCAM operation. The campaign is generally considered successful, contributing to an increase in the early detection of breast cancer with mammograms [3,4,5]. In the meantime, other cancer-related organizations established similar health observances. The American Lung Association’s LUNG FORCE initiative launched the Lung Cancer Awareness Month (LCAM), which started as Lung Cancer Awareness Day, in November [6], while the Prostate Cancer Foundation made September the Prostate Cancer Awareness Month (PCAM) in 1999 [7]. Each organization utilizes a variety of strategies, including social media, to promote cancer awareness, emphasize a focus on early detection, and provide educational materials to the public.

However, there have been no studies to evaluate the effectiveness of these health observances to improve public awareness using joinpoint regression analysis. The last study to analyze the effectiveness of BCAM was done more than 10 years ago using data from the early 2000s [8]. Given that internet searches have become a critical source of health-related information since then, it is valuable to revisit the question using up-to-data data. Also, online health information-seeking behavior has been used as a reliable surrogate of public attention or awareness [9]. Thus, this study aimed to evaluate whether BCAM, compared to LCAM and PCAM, has successfully increased public awareness of breast cancer (as compared to lung cancer or prostate cancer, respectively) in the U.S. using Google search data.

## 2. Materials and Methods

### 2.1. Data Source and Definition

Google Trends, a publicly available data source generated based on the total Google search data [10], has been utilized for social and behavioral health research [11,12,13,14,15,16,17,18,19,20,21,22,23,24]. This analysis enables us to determine the relative popularity of specific search terms in a particular category (for example, “health”), place, and time range, suggesting how popular the terms are at a specific time point. The relative popularity is noted as a relative search volume (RSV) on a scale of 0–100 (100 indicating the highest popularity) [11,15,16,17]. We used the volume of online information-seeking behaviors, including, but not limited to, cancer diagnosis, prevention, education, and product purchase, as a surrogate of public awareness [16].

### 2.2. Search Input

**Figure 1:**
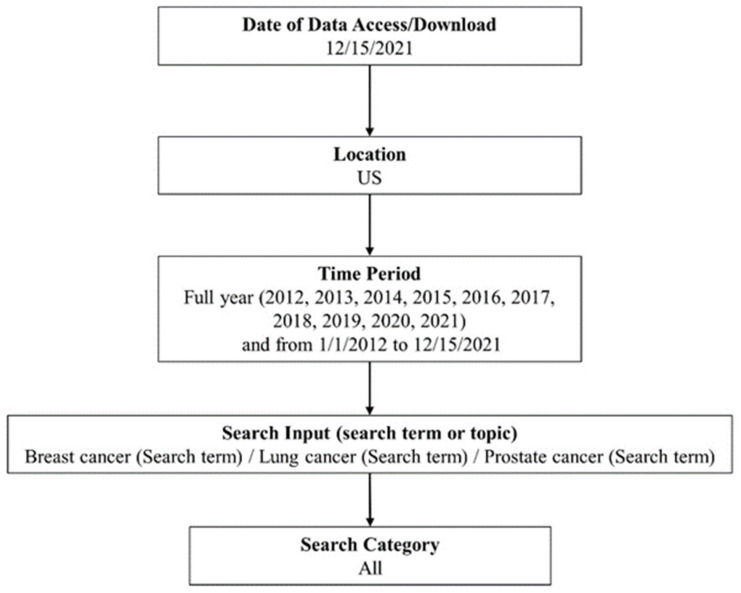
Google Trends search strategy.

Our initial search strategy using Google Trends is summarized in Figure 1 based on protocols reported in previous studies [13,15,17]. The search inputs included terms [Breast cancer], [Lung cancer], and [Prostate cancer]. We designated the U.S. as the location of the search. To categorize the three cancer-related searches further and to see the search trend for cancer in general, we performed additional searches using the search inputs [Cancer] and those including either breast, lung, or prostate cancer and donation, event, diagnosis, prevention, treatment, education, or product ([Breast cancer donation], for example). The strategy is summarized in Figure S1.

### 2.3. Search Variables

All searches were conducted with a “Search Term” option in all categories to extract the popularity of each search input (with a “Topic” option, search volumes of subtopics or relevant themes were also included). We selected time scales for each year (from 2012 to 2021) and a 120-month period (January 2012 to December 2021) to visualize the weekly and monthly trends of the RSVs. Based on a full-year analysis, we obtained weekly RSVs (each year contains 52 or 53 weeks; of note, PCAM (September) begins in the 35th or 36th week, BCAM (October) begins in the 39th or 40th week, and LCAM (November) begins in the 43rd or 44th week of the year). In the 120-month search, monthly aggregated RSVs were analyzed.

### 2.4. Statistical Analyses

We chose a joinpoint regression model using the Joinpoint Regression Program (version 4.9.0.0, March 2021, Statistical Research and Applications Branch, National Cancer Institute, MD, USA) to analyze time trends in the Google Trends RSV data [25]. Using this software, we identified time points, called joinpoints, where significant temporal trend changes occur. The analysis criteria were set to identify up to three joinpoints. The weekly or monthly percent changes (WPCs or MPCs) between trend-change points were determined with 95% confidence intervals (CIs). A *p*-value of <0.05 is defined as the threshold for statistical significance, indicating the level where the slope differs from zero.

### 2.5. Ethical Approval

We used the publicly available data published by Google Trends (Google LLC, Mountain View, CA, USA). The study was approved by the institutional review board of Okayama University Hospital with a waiver for informed consent since the study intended to analyze open data retrospectively (No. 1910-009). All research methods were performed following relevant guidelines and regulations.

## 3. Results

### 3.1. Trends in the Search Volume of the Term “Breast Cancer”

**Figure 2:**
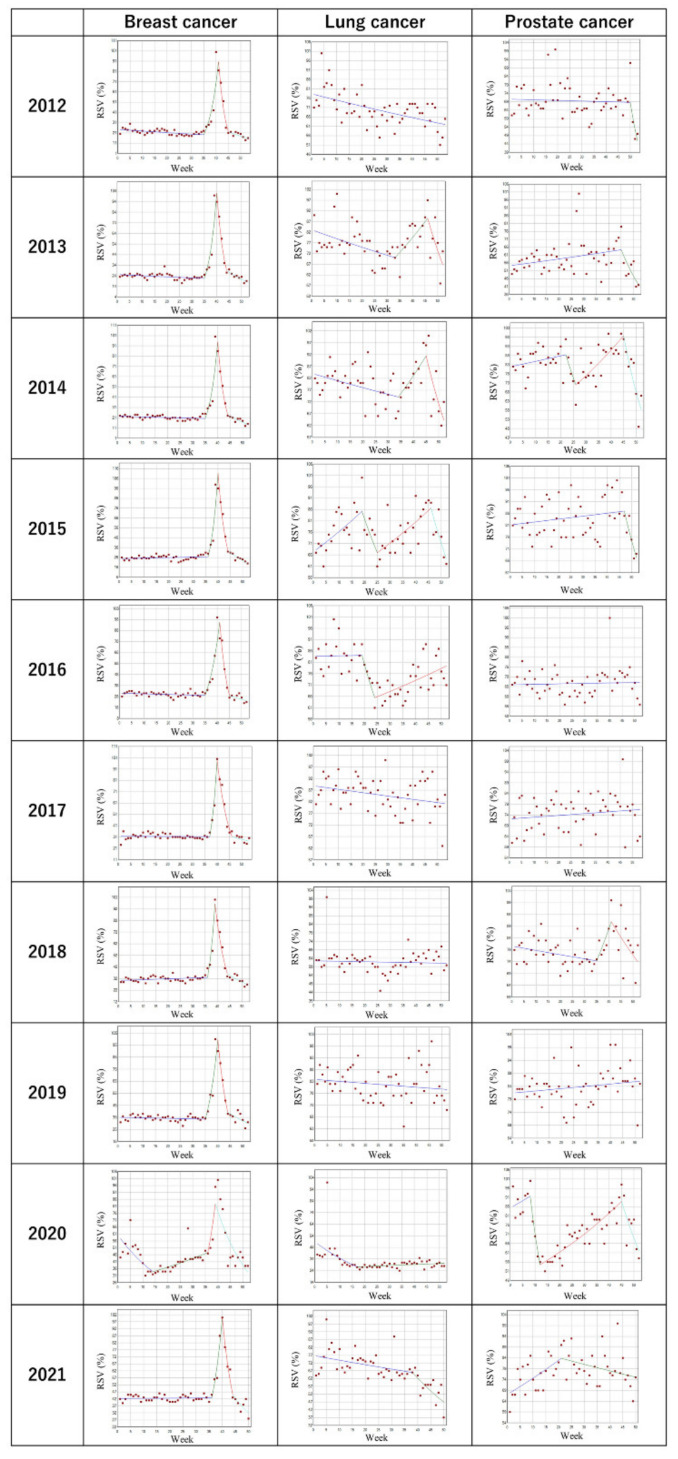
Trends in the weekly relative search volumes of “Breast cancer”, “Lung cancer”, and “Prostate cancer” in the United States (2012–2021). Weekly relative search volumes (RSVs) for the search terms “Breast cancer”, “Lung cancer”, and “Prostate cancer” are described. World Antimicrobial Awareness Week (WAAW) occurred in the 47th week of 2015, 2016, 2018, 2019, and 2020 and the 46th week of 2017. In the search results of the worldwide analysis in 2017 and 2020, the third joinpoints were identified around the time of WAAW (during the 48th week and 47th week, respectively). The number of slopes is determined by the number of joinpoints identified by the analysis. Joinpoints are the time points when statistically significant changes in the linear slopes are noted.

Table 1 and Figure 2 show the trends and changes in the weekly RSVs for the search term “Breast cancer” in the U.S. in each full year from 2012 to 2021. There was a significant increase in the RSV every year from around the 35th to 37th week toward the 39th to 41st week (the 1st or 2nd week of BCAM, *p* > 0.001), except for in 2020, when the increase in the RSV was 20.3% per week from the 36th to 39th week, without statistical significance. The WPC ranged from 21.9% to 46.7% during the same period. The increases in the RSV were followed by a significant decrease in the RSV every year (*p* < 0.001), with the third joinpoints at the 44th to 46th week, except for in 2020, when the third joinpoint was at the 39th week, followed by a significant decrease in the RSV by 6.7% per week (*p* < 0.001).

### 3.2. Trends in the Search Volume of the Term “Lung cancer”

Table 2 and Figure 2 describe trends and changes in the weekly RSVs for the search term “Lung cancer” in the U.S. in each full year from 2012 to 2021. There were no consistent trend changes observed in the case of lung cancer. In 2013 and 2015, there was a joinpoint during LCAM at the 46th week, with significant increases in the RSV at 1.8% and 1.2% per week, with following significant decreases in the RSV toward the end of the year at 4.6% and 4.8%, respectively (*p* < 0.05). However, there were no other joinpoints associated with lung cancer around the time of LCAM.

### 3.3. Trends in the Search Volume of the Term “Prostate Cancer”

Table 3 and Figure 2 describe trends and changes in the weekly RSVs for the search term “Prostate cancer” in the U.S. in each full year from 2012 to 2021. There were multiple points with trend changes; however, no joinpoints were found around the time of PCAM for prostate cancer, except for 2018, when the first joinpoint was observed at the 35th week with a following nonsignificant increase in the RSV at 3.4% per week through the 41st week.

### 3.4. Monthly Trends in RSVs between 2012 and 2021

**Figure 3:**
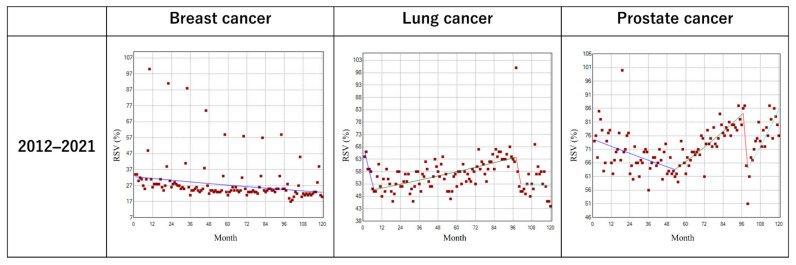
The trends in monthly relative search volumes between 2012 and 2021 with relevant search terms. Monthly RSVs for the search terms “Breast cancer”, “Lung cancer”, and “Prostate cancer” between 2012 and 2021 are shown.

Table 2 and Table 3, Supplementary Table S1, and Figure 3 show the monthly trends and trend changes in RSVs between 2012 and 2021 for the search terms “Breast cancer,” “Lung cancer”, and “Prostate cancer”. No joinpoints were observed with a monthly analysis from 2012 to 2021 for “Breast cancer”. Regarding “Lung cancer”, there was a modest but significant increase in the RSV from the 7th (July 2012) to 98th (February 2020) month at 0.3% monthly, with a following nonsignificant yet steep decrease in the RSV at 6.0% per month. For “Prostate cancer”, there was a significant increase in the RSV from the 53rd (May 2016) to 97th (January 2020) month at 0.6% monthly, with a following nonsignificant but considerable decrease in the RSV at 8.7% per month. Table S1 describes the average monthly trends during the period. Concerning the search result for “Breast cancer”, there was a significant continuous decrease in the RSV with the average MPC by 0.3% (*p* < 0.001). For the search results for “Lung cancer” and “Prostate cancer”, there were nonsignificant decreases in the RSV with the average MPCs by 0.2% and 0.1%, respectively.

### 3.5. Trends in the Search Volume of the Term “Cancer”

Figure S2 describes trends and changes in the monthly trends of RSVs for the term “Cancer” in the U.S. in each full year from 2012 to 2021. There were multiple points with trend changes, one of which was in the 97th month (January 2020), with a following nonsignificant decline in the RSV. However, the average MPC throughout the period was 0% (95% confidence interval (CI), −0.5 to 0.6).

### 3.6. Monthly Trends in RSVs for Subcategorical Analysis between 2012 and 2021

Supplementary Figures S3–S5 show the monthly trends and trend changes in RSVs between 2012 and 2021 for the search terms comprising “Breast cancer”, “Lung cancer”, or “Prostate cancer”, with “Donation”, “Event”, “Diagnosis”, “Prevention”, “Treatment”, “Education”, or “Product”. In breast-cancer-related searches, there were surges in the RSVs every October for the search terms “Breast cancer donation”, “Breast cancer event”, “Breast cancer prevention”, “Breast cancer treatment”, and “Breast cancer education”. However, the average MPCs during the period for these search terms were within ±0.1% in searches for donation, diagnosis, or treatment. There were negative trends for event, prevention, and education, with average MPCs of −1.0% (95% CI −1.3 to −0.6, *p* < 0.001), −0.7% (95% CI −2.7 to 1.4, *p* = 0.507), and −0.3% (95% CI −0.5 to −0.1, *p* = 0.002), respectively. The average MPC for “Breast cancer product” was 0.5% (95% CI −0.2 to 1.2, *p* = 0.132).

For lung-cancer-related searches, there were no surges in the RSVs during the months of LCAM. No trend changes during the period were noted for “Lung cancer donation”, “Lung cancer diagnosis”, or “Lung cancer prevention”. For “Lung cancer event”, “Lung cancer treatment”, “Lung cancer education”, and “Lung cancer product”, the average MPCs were 1.9% (95% CI 0.8–3.0, *p* = 0.001), 0.1% (95% CI 0–0.3, *p* = 0.047), 1.2% (95% CI 0.5–1.8, *p* < 0.001), and 3.6% (95% CI 2.4–4.8, *p* < 0.001), respectively.

For prostate cancer, there were insufficient search volumes for “Prostate cancer donation”, “Prostate cancer event”, and “Prostate cancer product” during the period to run Google Trends searches. No surges in the RSVs around PCAM were observed in any of the search terms. There were no trend changes based on average MPCs during the period for “Prostate cancer diagnosis”, “Prostate cancer prevention”, “Prostate cancer treatment”, or “Prostate cancer product”.

## 4. Discussion

This study evaluated how BCAM has affected public awareness of breast cancer, as compared to LCAM and PCAM for lung and prostate cancer, the most common cancers in the U.S., using the RSV as a surrogate. Compared to the previous study that employed analysis of variance and was performed when Google Trends was not yet available, our study successfully showed the effectiveness of BCAM via joinpoint regression analysis, an accurate method used to assess changes in time series data [26]. Also, we performed subcategory searches to interpret the overall search trends better. Our results showed that BCAM had significant impacts on the U.S. public interest in breast cancer from 2012 to 2021, with peaks in the RSVs during the BCAM periods, while LCAM and PCAM did little to affect the public interest in lung or prostate cancer. The findings imply that BCAM may have effectively improved public awareness of breast cancer. Of note, the RSVs for breast, lung, and prostate cancers in the U.S. shot up around the 15th week of 2020, when the COVID-19 pandemic began, although BCAM continued to be associated with increasing RSVs of breast cancer in 2020, unlike the other two awareness months. The dramatic increases in the RSVs for the cancers could be related to concerns among the general public that those who are immunocompromised might be more vulnerable to COVID-19, as seen in the data set in inflammatory bowel diseases [24]. However, contrasting data from Canada showed that interest in various cancers temporarily decreased during the early pandemic [27]. Also, the overall RSVs for cancer in the U.S. trended downward in early 2020 (Figure S2). Further studies are warranted to observe changes in the global public interest regarding cancers during the pandemic.

What makes BCAM successful at stimulating public awareness? Based on the results of subcategorical analysis, search volumes for breast cancer donation, events, prevention, treatment, and education increased in association with BCAM, which suggests that BCAM may have triggered public interest in breast cancer prevention and fundraising. Given the complexity and the issues associated with cancer awareness campaigns, multidisciplinary approaches involving the various foci listed above are crucial [28]. Two other possible explanations include increased funding focused on marketing and the strategic use of the pink ribbon symbol. Based on financial statements of the National Breast Cancer Foundation (NBCF), American Lung Association (ALA), and Prostate Cancer Foundation (PCF), the ALA had the highest total liabilities and net assets in the fiscal year of 2020 (FY20) with USD 196,583,744, followed by PCF at USD 43,134,308 and NBCF at USD 6,157,834 [29,30,31]. However, looking at their expenses, NBCF spent 69% of its total expenses on education and outreach in FY20, a total of USD 9,081,403, as compared to ALA, which spent 14% of its total expenses on advocacy, and PCF, which spent 13% on public awareness. The NBCF’s focus on public awareness, which is reflected in their high percentages of financial expenditure on education and outreach, could have resulted in the success of BCAM. The relationship between each site-specific cancer organization’s expenditure and public awareness needs to be clarified in future research.

All the organizations have utilized social media, an important tactic to disseminate information, and shared educational materials on their websites. A recent systematic scoping review showed that social media promotion may facilitate behavioral changes related to cancer prevention, but the evidence for this is still limited [32]. Unlike the other two cancers, breast cancer awareness has also been driven by the pink ribbon movement, a symbol representing support of breast cancer care patients and survivors [33]. The subcategorical analysis results also suggested that the RSVs for breast cancer donations, events, and prevention temporarily surged in response to BCAM. Pink ribbons have been effectively utilized as a symbol to promote breast cancer awareness, often in association with commercialized products. The involvement of non-medical partners, accounting for more than 120 organizations in 2020 [34], and influencers affected by breast cancer, might have impacted BCAM’s effectiveness in October. It is conceivable that more partners resulted in more advertisements seen in our daily lives [35].

Public awareness of breast cancer is considered favorable and is credited with decreasing breast cancer mortality by up to 20% due to early detection via mammograms [36]. However, the increase in breast cancer awareness and popularity of mammograms is also associated with harm, including false-positive test results and overdiagnosis [37]. The U.S. Preventive Services Task Force recommends that the decision to proceed with mammography for women aged 40 to 49 years be made based on clinical considerations of the woman and her preferences to balance the benefits and harms [38]. However, awareness about the overdiagnosis of breast cancer may not be sufficient in the general public [39]. Given the time constraints in primary care settings, information about mammography’s harms may not always be provided to patients, especially those with limited health literacy [40].

Our study quantified the extent of public awareness of breast, lung, and prostate cancers in the U.S. using joinpoint regression analysis and Google Trends as the data source, which enabled us to visualize the trends related to the relevant health observances. However, there are limitations to be addressed. First, the results only included those with internet access and seeking health information via a Google search. Given the high internet penetration rates in the U.S., at around 90.4% [41], and the high market share of Google search, at 83% [42], Google Trends may be considered a good surrogate of public awareness. Second, the changes in RSVs could be affected by news or media coverage associated with the disease, which decreases the certainty that we can attribute the changes in RSVs to a particular phenomenon such as BCAM. Third, because Google Trends data are an indirect measure of public engagement, direct measures, including large-scale surveys, may be beneficial to clarify the findings. Also, it needs to be noted that there may be several fundraising groups other than ALA, NBCF, and PCF contributing to the advocacy of these malignancies. Despite these limitations, we believe that our approach satisfactorily reveals the trends in public awareness for breast, lung, and prostate cancers.

## 5. Conclusions

In conclusion, the results of this study suggest that BCAM may have successfully improved public awareness of breast cancer in the U.S., as compared to LCAM and PCAM for lung and prostate cancers. While high awareness has been criticized for contributing to the overdiagnosis of breast cancer, enhancing public awareness is recognized as a crucial measure to alleviate the disease burden of cancers. The success of BCAM is likely related to multiple factors, including its potential effect to facilitate behavioral changes related to cancer prevention, aggressive marketing strategy, effective involvement of non-medical industries, involvement of influencers affected by breast cancer, and awareness symbol. The effectiveness of BCAM at raising public awareness could serve as an example for organizations working on health observances or awareness campaigns.

## Figures and Tables

**Table 1:** Trend changes in relative search volumes of the term “Breast cancer” (2012–2021).

Term	Year	Period 1		Period 2		Period 3		Period 4	
		Weeks	WPC (%) [95% CI]	Weeks	WPC (%) [95% CI]	Weeks	WPC (%) [95% CI]	Weeks	WPC (%) [95% CI]
Breast cancer	2012	1–35	−0.7 * [−1.1, −0.2]	35–41	29.5 * [19.2, 40.6]	41–45	−29.7 * [−41.5, −15.4]	45–53	−4.1 * [−7.8, −0.1]
	2013	1–35	−0.5 * [−0.9, 0]	35–40	36.6 * [22.8, 52.0]	40–44	−27.1 * [−38.4, −13.7]	44–52	−5.4 * [−8.8, −1.9]
	2014	1–35	−0.3 [−0.7, 0.1]	35–40	36.2 * [22.7, 51.2]	40–44	−29.2 * [−40.0, −16.5]	44–52	−6.0 * [−9.3, −2.5]
	2015	1–35	0.2 [−0.1, 0.6]	36–40	42.7 * [23.9, 64.4]	40–44	−26.2 * [−35.9, −15.0]	44–52	−6.0 * [−8.8, −3.0]
	2016	1–35	−0.2 [−0.5, 0.1]	35–41	21.9 * [15.8, 28.3]	41–45	−25.9 * [−33.9, −17.0]	45–52	−2.4 [−5.3, 0.6]
	2017	1–37	0 [−0.3, 0.2]	37–40	46.7 * [15.6, 86.2]	40–46	−17.5 * [−21.8, −13.0]	46–53	−1.9 [−5.0, 1.3]
	2018	1–36	0.2 [0, 0.5]	36–39	43.0 * [16.6, 75.5]	39–44	−17.8 * [−23.0, −12.4]	44–52	−3.6 * [−5.7, −1.4]
	2019	1–35	−0.1 [−0.4, 0.2]	35–40	24.3 * [14.7, 34.7]	40–44	−20.9 * [−30.3, −10.1]	44–52	−3.2 * [−5.8, −0.5]
	2020	1–14	−4.0 [−6.3, −1.7]	14–36	1.6 * [0.4, 2.8]	36–39	20.3 [−24.1, 90.4]	39–52	−6.7 * [−8.9, −4.4]
	2021	1–36	0 [−0.2, 0.3]	36–40	23.6 * [11.8, 36.6]	40–44	−18.1 * [−25.9, −9.5]	44–50	−5.4 * [−8.6, −2.2]

* Significantly different from zero (*p* < 0.05). Abbreviations: CI, confidence interval; WPC, weekly percentage change. Periods were separated as Periods 1–4, when the trend changes were statistically detected in the Joinpoint regression analysis during the study period. For each year’s results, weekly percentage changes are shown.

**Table 2:** Trend changes in relative search volumes of the term “Lung cancer” (2012–2021).

Term	Year	Period 1		Period 2		Period 3		Period 4	
		Weeks/Months	WPC (%)/MPC (%) [95% CI]	Weeks/Months	WPC (%)/MPC (%) [95% CI]	Weeks/Months	WPC (%)/MPC (%) [95% CI]	Weeks/Months	WPC (%)/MPC (%) [95% CI]
Lung cancer	2012	1–53	−0.4 * [−0.6, −0.3]						
	2013	1–33	−0.5 * [−0.9, −0.1]	33–46	1.8 * [0.2, 3.5]	46–52	−4.6 * [−9.0, 0]		
	2014	1–34	−0.4 [−0.7, 0]	34–45	1.9 [−0.1, 4.0]	45–52	−4.9 * [−8.1, −1.6]		
	2015	1–19	1.2 * [0.4, 2.1]	19–25	−3.9 [−9.3, 1.7]	25–46	1.2 * [0.5, 1.9]	46–52	−4.8 * [−8.9, −0.6]
	2016	1–19	0 [−0.7, 0.8]	19–24	−4.9 [−11.7, 2.5]	24–52	0.7 * [0.3, 1.1]		
	2017	1–53	−0.2 [−0.3, 0]						
	2018	1–52	0 [−0.2, 0.2]						
	2019	1–52	−0.1 [−0.3, 0.1]						
	2020	1–17	−3.4 * [−5.0, −1.7]	17–52	0.3 [−0.2, 0.8]				
	2021	1–38	−0.4 * [−0.7, −0.1]	38–50	−2.8 * [−4.6, −1.0]				
	2012–2021	1–7	−4.4 * [−7.9, −0.7]	7–98	0.3 * [0.2, 0.3]	98–101	−6.0 [−24.7, 17.3]	101–120	−0.1 [−0.7, 0.6]

* Significantly different from zero (*p* < 0.05). Abbreviations: CI, confidence interval; MPC, monthly percentage change; WPC, weekly percentage change. Periods were separated as Periods 1–4, when the trend changes were statistically detected in the Joinpoint regression analysis during the study period. For the results from 2012 to 2021, monthly percentage changes are shown. For each year’s results, weekly percentage changes are shown.

**Table 3:** Trend changes in relative search volumes of the term “Prostate cancer” (2012–2021).

Term	Year	Period 1		Period 2		Period 3		Period 4	
		Weeks/Months	WPC (%)/MPC (%) [95% CI]	Weeks/Months	WPC (%)/MPC (%) [95% CI]	Weeks/Months	WPC (%)/MPC (%) [95% CI]	Weeks/Months	WPC (%)/MPC (%) [95% CI]
Prostate cancer	2012	1–50	0.1 * [−0.3, 0.2]	50–53	−12.7 [−26.2, 3.3]				
	2013	1–45	0.4 * [0, 0.7]	45–52	−5.9 * [−10.9, −0.6]				
	2014	1–22	0.3 [−0.3, 1.0]	22–26	−5.0 [−16.6, 8.1]	26–45	1.7 * [0.8, 2.5]	45–52	−7.2 * [−10.4, −4.0]
	2015	1–47	0.1 [−0.1, 0.3]	47–52	−5.0 [−10.3, 0.6]				
	2016	1–52	0 [−0.1, 0.2]						
	2017	1–53	0.1 [−0.1, 0.3]						
	2018	1–35	−0.2 [−0.5, 0]	35–41	3.4 [−1.5, 8.6]	41–52	−1.9 * [−3.3, −0.4]		
	2019	1–52	0.1 [0, 0.3]						
	2020	1–8	0.9 [−2.4, 4.3]	8–12	−12.2 * [−22.4, −0.6]	12–45	1.5 * [1.2, 1.8]	45–52	−5.0 * [−8.1, −1.8]
	2021	1–21	1.1 * [0.3, 1.8]	21–50	−0.4 * [−0.8, 0]				
	2012–2021	1–53	−0.3 * [−0.4, −0.2]	53–97	0.6 * [0.5, 0.8]	97–100	−8.7 [−25.6, 11.9]	100–120	1.5 * [0.9, 2.0]

* Significantly different from zero (*p* < 0.05). Abbreviations: CI, confidence interval; MPC, monthly percentage change; WPC, weekly percentage change. Periods were separated as Periods 1–4, when the trend changes were statistically detected in the Joinpoint regression analysis during the study period.

## Data Availability

The data presented in this study are available on request from the corresponding author. The data presented in this study are openly available in FigShare at https://doi.org/10.6084/m9.figshare.19778899 (accessed on 17 May 2022).

## Supplementary Materials

The following supporting information can be downloaded at: https://www.mdpi.com/article/10.3390/cancers14102534/s1, Figure S1: Additional Google Trends search strategy; Figure S2: The trends in the monthly relative search volume of “Cancer” between 2012 and 2021; Figure S3–S5: The trends in the monthly relative search volumes of “Breast cancer”, “Lung cancer”, and “Prostate cancer”, in different subcategories.
