# Supplementary material for: Impact of Breast Cancer Awareness Month on Public Interest in the United States between 2012 and 2021: A Google Trends Analysis
Source: Cancers (Basel). 2022 May 21;14(10):2534. doi: 10.3390/cancers14102534 (PMC9140129; doi:10.3390/cancers14102534)
Supplement: Supplementary file 1 [file cancers-14-02534-s001.zip › cancers-1719887-Supplementary.pdf]

Supplementary materials

Impact of Breast Cancer Awareness Month on Public Interest in the United States between 2012 and 2021: A Google Trends Analysis

Yoshito Nishimura and Jared D. Acoba

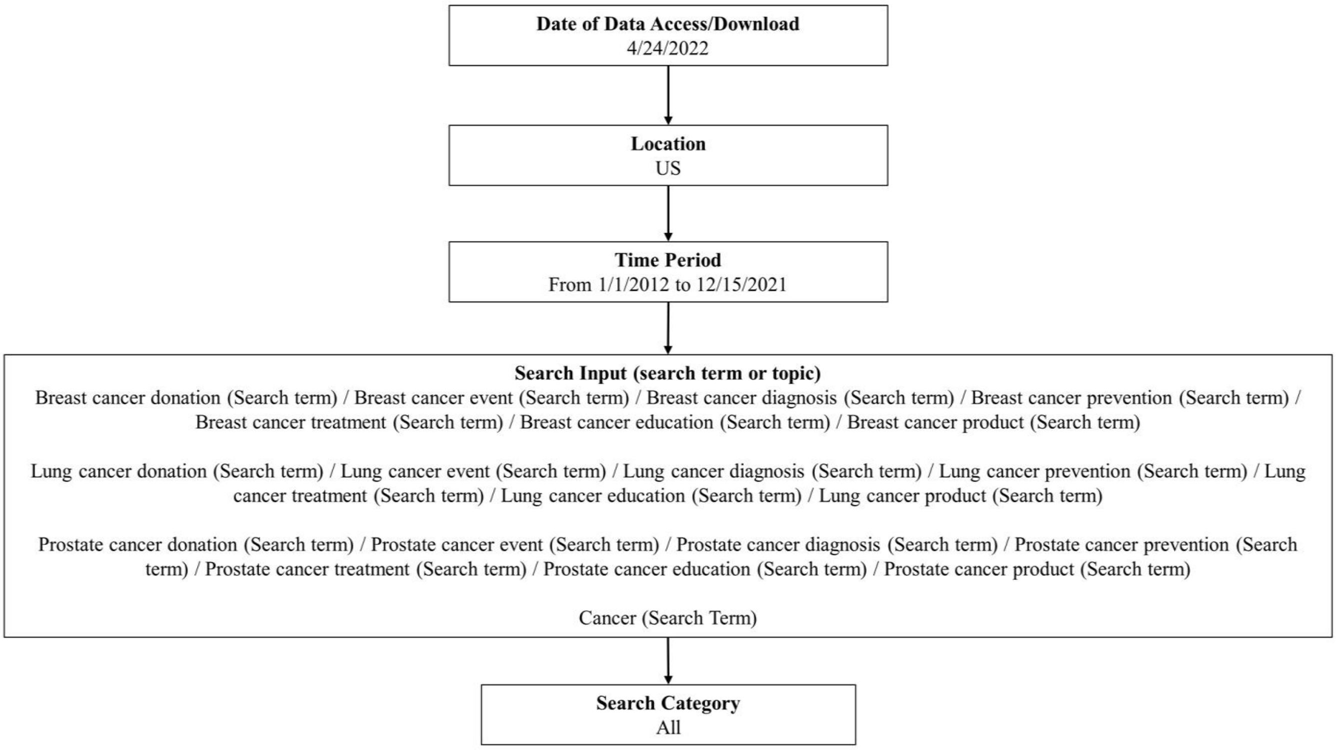

Figure S1. Additional Google Trends search strategy.

	Cancer
2012-2021	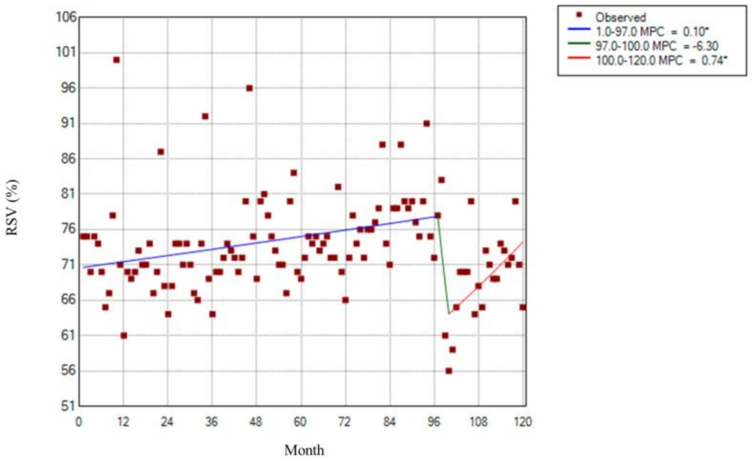

Figure S2. The trends in the monthly relative search volume of "Cancer" between 2012 and 2021.

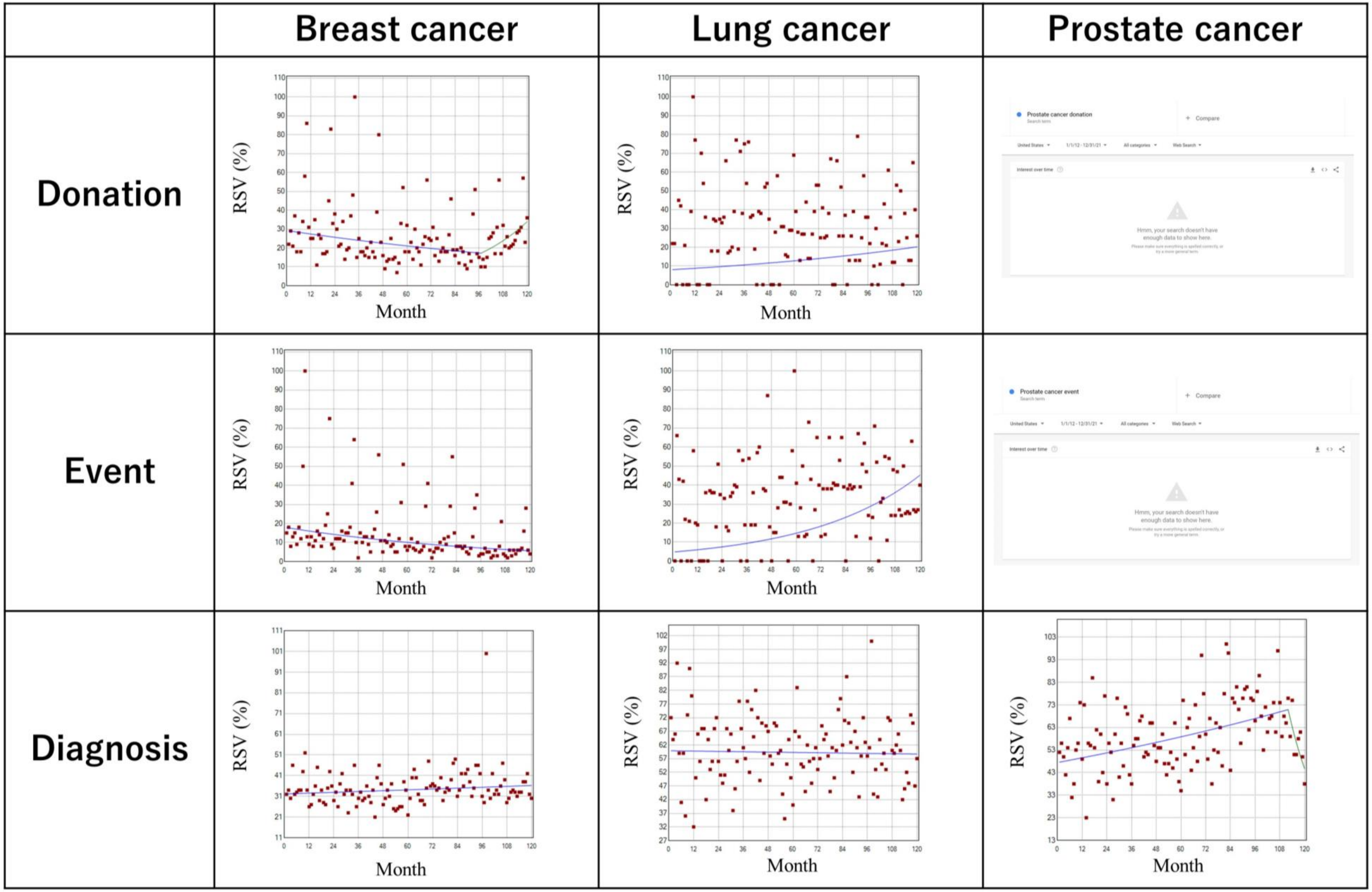
Breast cancer
Lung cancer
Prostate cancer
Donation
Event
Diagnosis
RSV (%)
Month
Prostate cancer donation
Compare
Hmm, your search doesn't have enough data to show here.
Prostate cancer event

Figure S3

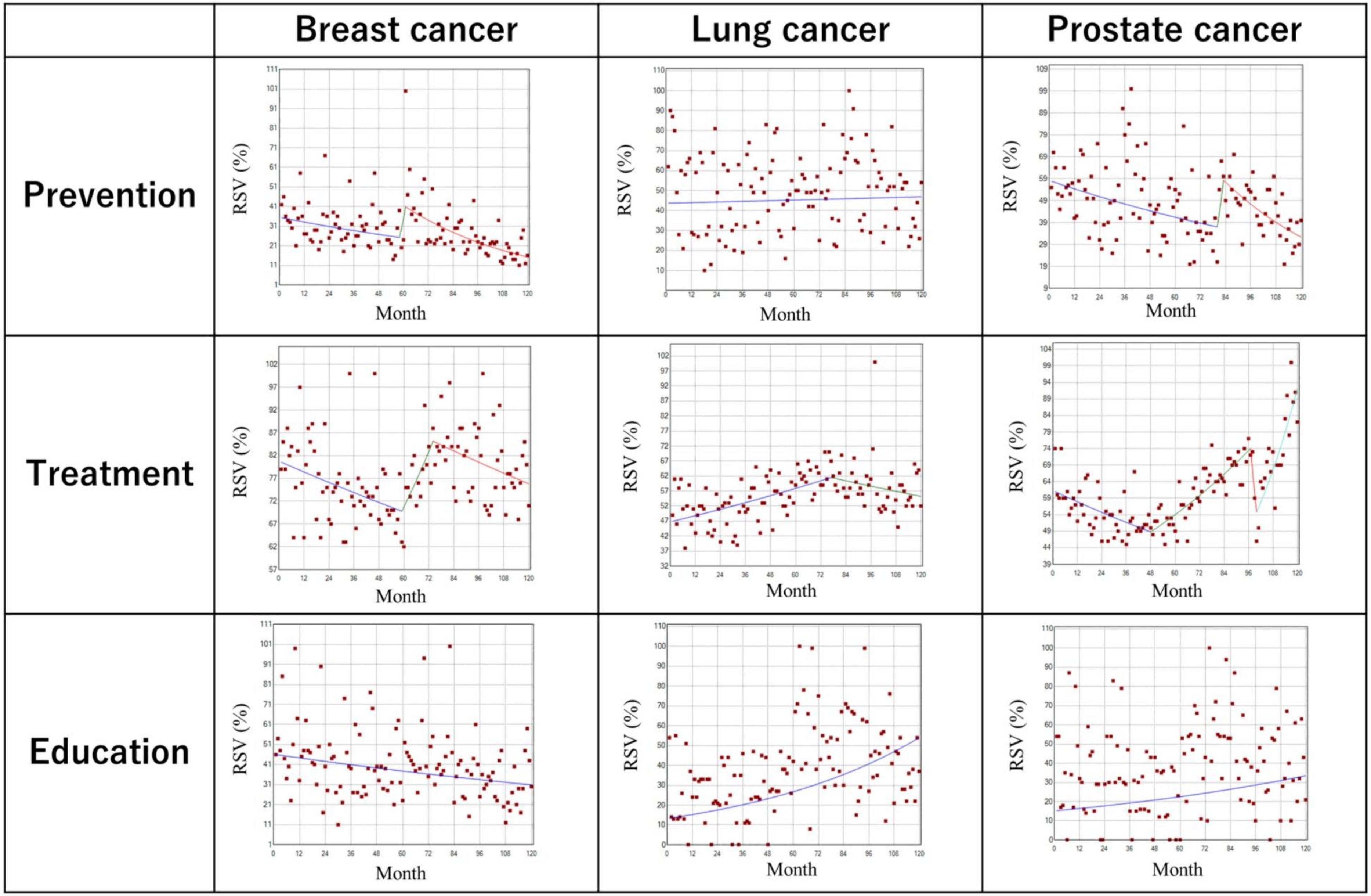
Breast cancer
Lung cancer
Prostate cancer
Prevention
Treatment
Education
RSV (%)
Month

Figure S4

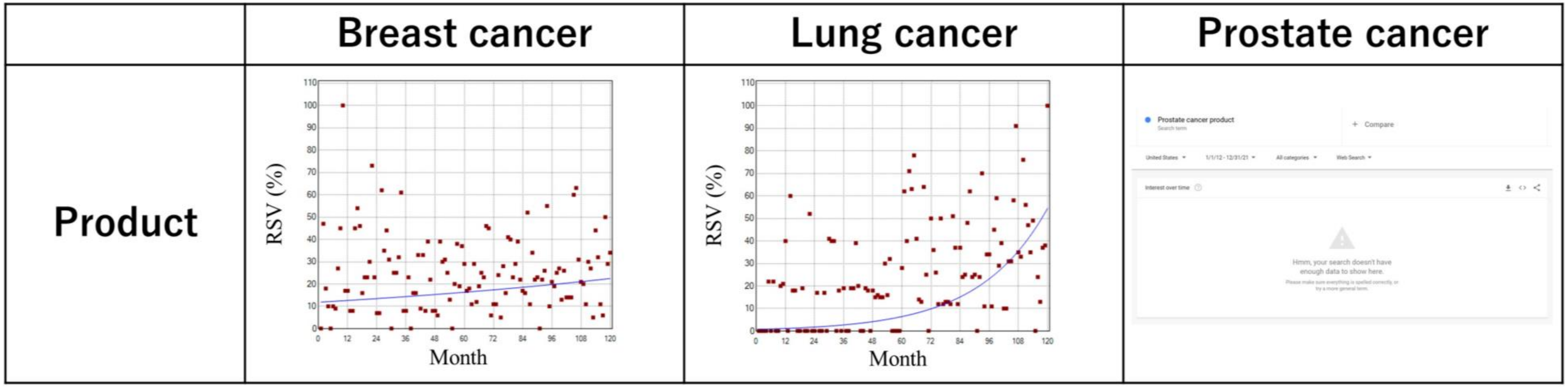

Figure S5

Figure S3–S5. The trends in the monthly relative search volumes of "Breast cancer," "Lung cancer," and "Prostate cancer," in different subcategories.

Table S1. Average monthly trends in relative search volumes of relevant search terms [2012–2021].

Word	Average MPC (%) [95% CI]
Breast cancer	-0.3* [-0.5, -0.1]
Lung cancer	-0.2 [-0.8, 0.4]
Prostate cancer	-0.1 [-1.2, 0.9]

* Significantly different from zero (P < 0.05). Abbreviations: CI, confidence interval; MPC, monthly percentage change. Average monthly percentage changes are shown from month 1 (January 2012) to month 120 (December 2021).
